# Cognitive Behaviour Therapy Versus a Counselling Intervention for Anxiety in Young People with High-Functioning Autism Spectrum Disorders: A Pilot Randomised Controlled Trial

**DOI:** 10.1007/s10803-017-3252-8

**Published:** 2017-08-02

**Authors:** Suzanne M. Murphy, Uttom Chowdhury, Susan W. White, Laura Reynolds, Louisa Donald, Hilary Gahan, Zeinab Iqbal, Mahesh Kulkarni, Louise Scrivener, Hadi Shaker-Naeeni, Dee A. Press

**Affiliations:** 1 Institute for Health Research, University of Bedfordshire, University Square, Luton, Bedfordshire LU1 3JU UK; 2 East London NHS Foundation Trust, Specialist Child and Adolescent Mental Health Services, Beech Close Resource Centre, Beech Road, Dunstable, Bedfordshire LU6 3SD UK; 3 Department of Psychology, Virginia Polytechnic Institute and State University, Blacksburg, VA USA; 4 Faculty of Health Sciences, Southampton University, University Road, Southampton, SO17 1BJ UK; 5 Huntercombe Hospital, Buxton, Norwich, NR10 5RH UK

**Keywords:** Randomised controlled trial, Cognitive behavioural therapy, Counselling, Autism spectrum disorder, Anxiety, Adolescent

## Abstract

The use of cognitive-behavioural therapy (CBT) as a treatment for children and adolescents with autism spectrum disorder (ASD) has been explored in a number of trials. Whilst CBT appears superior to no treatment or treatment as usual, few studies have assessed CBT against a control group receiving an alternative therapy. Our randomised controlled trial compared use of CBT against person-centred counselling for anxiety in 36 young people with ASD, ages 12–18. Outcome measures included parent- teacher- and self-reports of anxiety and social disability. Whilst each therapy produced improvements in participants, neither therapy was superior to the other to a significant degree on any measure. This is consistent with findings for adults.

## Introduction

Elevated levels of anxiety frequently manifest in young people with autism spectrum disorder (ASD; Vasa et al. 2014). A recent meta-analysis (van Steensel et al. 2011) reported that nearly 40% of children with ASD reached clinical thresholds for at least one anxiety disorder. For typically developing children and adolescents, cognitive-behavioural therapy (CBT) is the recommended treatment and systematic reviews have reported its efficacy (Higa-McMillan et al. 2016). A recent systematic review of psychotherapies for anxiety for typically-developing children (Reynolds et al. 2012) concluded that the current, dominant therapy is CBT with the vast majority of trials evaluating this approach.

Fewer trials *specifically* for children with ASD are available, but the most recent meta-analysis (Ung et al. 2015) found a moderate overall effect size in favour of CBT. However, a recent Cochrane review (James et al. 2015), including trials for *both* typically developing children and those with ASD, highlighted differences in the relative effectiveness of CBT when tested against ‘active’ or ‘passive’ control groups. James et al. (2015) defined ‘passive’ control groups as not receiving any treatment during the period of assessment (typically a wait list). By contrast, they defined ‘active’ controls as procedures (attention placebos) controlling for non-specific treatment factors (e.g., contact time and attention) but not consisting of specific treatments for anxiety, such procedures included bibliotherapy, psychoeducation, emotional disclosure, therapist and peer support. In addition to active attention controls, a few studies compared CBT to treatment as usual (comprising a mix of interventions and approaches) and one study (Sung et al. 2011) compared CBT to an alternative social recreation treatment. James et al. (2015) found CBT to be significantly more effective than passive controls in reducing symptoms of anxiety; this was consistent with earlier reviews. However, it was no more effective than *non-CBT active* control treatments or treatment as usual. The authors concluded that there now exist sufficient robust CBT trials with passive controls; however, comparisons between CBT and active controls and head to head comparisons with alternative treatments are lacking. Use of treatment as usual as a comparison is not without problems; being highly variable in intensity, frequency, duration and type, it therefore may not match a CBT standardised intervention for therapist contact time and attention. Indeed, one such study (Storch et al. 2013) acknowledged that 25% of their ‘treatment as usual’ control group in fact received no treatment at all. To provide a valid control group and address the limitations inherent with treatment as usual comparisons, the study described here compares CBT for anxiety in young people with ASD to a consistently-applied alternative therapy (person-centred counselling).

Within the National Health Service (NHS) in the United Kingdom (UK), a commonly-offered alternative to CBT or drug therapy is counselling for those who do not wish to use, or are unsuitable for, these approaches. Counselling as currently offered by the NHS largely centres on person-centred therapy, also called non-directive therapy (Gibbard and Hanley 2008). There is less available evidence to support its effectiveness than CBT and most of the extant research relates to adults rather than children. A recent systematic review for anxiety treatment in typically developing children (Higa-McMillan et al. 2016) identified only one study involving counselling; Andrews (1971) found that for adolescent boys, client-centred counselling was less effective than a behavioural intervention. For adults, a little more evidence is available. A Cochrane review (Bower et al. 2011) of counselling for mental health problems for adults found significantly greater short-term clinical effectiveness compared with usual care. A few trials and reviews, with adult samples, have compared CBT and to counselling head-to-head (Barrowclough et al. 2001; Cape et al. 2010; King et al. 2013; Morrell et al. 2009) all have found CBT and counselling to be equally effective. We report here the first pilot study to undertake a comparison of CBT against counselling for children or adolescents with ASD. The primary aim of this study was to compare CBT against a counselling intervention for anxiety in young people with ASD. A secondary aim was to compare outcomes for social skills between these two interventions as the CBT package used included a social skills component. We did not frame specific hypotheses as to the direction of these differences because, as yet, no previous studies have compared these interventions in this client group.

## Method

### Design

Study design was a pilot randomised controlled trial (RCT) with two study arms; a CBT intervention, the Multimodal Anxiety and Social Skill Intervention for adolescents with ASD (MASSI, White et al. 2010) versus counselling as offered by the NHS in the UK. Assessments were performed at baseline, within 4 weeks of completion of treatment and at 12-week follow-up. Randomisation was managed by a statistician unconnected with the study who prepared sequentially numbered envelopes containing allocation status. All baseline, post-test and fidelity measures were collected by blind independent assessors.

### Participants

Participants were young people and their parents attending three NHS Child and Adolescent Mental Health Services (CAMHS) clinics in the South of England. The designated area of responsibility of NHS CAMHS clinics is to provide treatment for moderate to severe mental health problems up to and including 18 years of age. All participants aged 12–18 years old referred to the clinic between April 2011 and April 2013 with a diagnosis of ASD and anxiety were invited to participate.

The MASSI CBT programme was developed as a treatment for young people with ASD and anxiety. Participants were required to meet diagnoses based on the Autism Diagnostic Schedule (ADOS, Lord et al. 2002), the Autism Diagnostic Interview—Revised (ADI-R. Rutter et al. 2003) and the Anxiety Disorders Interview Schedule (ADIS, Silverman and Albano 1996). MASSI is designed for young people aged 12–17 with IQ > 70. In the area of the UK in which we were recruiting, children with ASD but without learning/intellectual disabilities (defined as IQ < 70) attend mainstream schools, whereas those with ASD co-occurring with learning disability generally attend special schools. Current or recent mainstream school attendance was therefore a study entry criterion. MASSI has not been designed to address obsessive–compulsive disorder (OCD), panic disorder, agoraphobia with/without panic disorder (PD/agor) and post-traumatic stress disorder (PTSD) and is not recommended for co-occurring problems such as psychosis, severe, untreated clinical depression or substance abuse. Therefore, patients with these primary diagnoses were excluded. Receipt of concurrent psychological therapy from another source (e.g., school, voluntary organisation, private treatment) also excluded young people from participation.

Participating staff providing the treatments were all employed by NHS CAMHS clinics and comprised three consultant child psychiatrists, one clinical psychologist and one counsellor. All were professionally trained in either CBT or counselling, and years of experience ranged from 3 to 21. Treating clinicians were requested not to alter medication doses during the period of intervention. Two therapists provided CBT only (four participants each) and two provided counselling only (five and six participants), the fifth therapist provided both counselling and CBT (nine CBT participants and eight counselling participants). All clinicians received supervision with peer group as part of their NHS work. The supervision was not specific to the study itself but part of their usual day to day practice.

### Measures

#### Outcome Measures

##### Anxiety Disorders Interview Schedule for Children/Parents (ADIS-C/P; Silverman and Albano 1996)

A review of trials investigating the use of CBT to treat anxiety in children and adolescents with ASD (Sukhodolsky et al. 2013), reported that ADIS had successfully been used with this population. ADIS was used both as a selection measure to confirm diagnosis and as a baseline and outcome measure. The parent and adolescent participants were interviewed separately as recommended by authors by an independent clinical evaluator who then assigned an overall rating of severity (Clinician Severity Rating; CSR) on a 9-point scale, with a score of four or more indicating a clinical diagnosis (Silverman and Albano 1996). A CSR of at least four for separation anxiety, social anxiety, specific phobia and/or generalised anxiety was required for participation.

The authors of ADI-R, ADOS and ADIS recommend administration by fully-trained clinicians; independent blind clinical evaluators for this study were a clinical psychologist of 11 years’ experience and a research assistant with clinical experience. Fidelity for ADI-R and ADOS was ensured as our assessors satisfactorily completed accredited courses including fidelity assessment. ADIS training for the assessors was conducted as described in Liber et al. (2010) and 15% of the ADIS-C/P diagnostic assessments were independently reviewed by a consultant child psychiatrist and a clinical psychologist experienced in diagnosis and treatment of anxiety disorders and ASD. Agreement between clinically-trained raters of ADIS, has been found to be excellent for youth with ASD (Ung et al. 2014) and consistently with this, inter-rater reliability between our assessors using Cohen’s Kappa (weighted) = 0.88 (kappa values ranging from 0.81 to 1.00 are rated as ‘almost perfect agreement’; Landis and Koch 1977). Due to the recommended method of interview administration (not all respondents are required to answer every item) it is not possible to use a test of internal consistency for ADIS.

##### Child and Adolescent Symptom Inventory-4 ASD Anxiety Scale (CASI-anx; Hallett et al. 2013)

Anxiety measurement can be difficult in young people with ASD as respondents struggle to distinguish between ASD symptoms and anxiety symptoms. CASI-anx has been developed specifically to measure anxiety in children with ASD, the advantage of using CASI-anx is that items directly related to ASD have been removed therefore the scale as a whole has little or no overlap with symptoms of ASD (Hallett et al. 2013). For this reason CASI-anx was included as a supplementary measure to ADIS. It has been validated on large samples of children with ASD showing high internal consistency (Cronbach’s α = 0.85) and low correlations with ASD symptoms (Hallett et al. 2013). For our sample, internal consistency was also high (Cronbach’s α = 0.90).

##### Social Responsiveness Scale (SRS, Constantino and Gruber 2005)

SRS is designed to measure the severity and type of social impairments in youth with ASD (Constantino and Gruber 2005). Studies indicate that SRS has very high internal consistency (Cronbach’s α = 0.97, Constantino and Gruber 2005). SRS is a measure of social disability, and the subscales Social Motivation and Social Communication were selected to assess the social skills elements (see below) of the interventions. Social functioning is often more visible to teachers than to parents, therefore both parent- and teacher-report versions of SRS were collected. For the present study, parent SRS Cronbach’s α = 0.82 and teacher SRS Cronbach’s α = 0.84.

#### Fidelity Measures

McArthur et al. (2012) point out that whilst some aspects of treatment fidelity are generally well-reported, such as clear details of the intervention and number and duration of sessions, other assessments, such as checks that clinicians adhere to the intervention and measures of non-specific intervention effects, such as therapeutic alliance, are rarely included. To address these issues, we included the Primary Care Therapy Process Rating Scale (PCTPRS) (Godfrey et al. 2007) to measure adherence to CBT/counselling and the Therapy Process Observational Coding System—Alliance scale (TPOCS-A, McLeod and Weisz 2005) to measure therapeutic alliance. Both these measures rely on video-assessment of the treatment sessions by blinded, independent raters. McLeod and Weisz (2005) have stressed the importance of such raters (as opposed to client’s self-ratings, or ratings from the treating clinician) to avoid possible bias. Therapists also were blind and did not know which sessions were to be used for assessment; to maintain blinding they video-recorded all sessions (except in a few instances were clients requested not to be recorded). The third session was rated for each participant, if the third session was not available, then the fourth or fifth session was used. This session was used following recommendations to measure alliance early in treatment; later measures can be confounded with general symptom improvement (Lerner et al. 2011).

Training for use of TPOCS-A and PCTPRS was given as per the manuals (Godfrey et al. 2007; McLeod and Weisz 2005). Full details of video-recording and training for fidelity rating for our study have been given in Brown et al. (2015). Inter-rater reliability was calculated as the agreement between the three raters who rated all videos; these were two post-graduate clinical psychologists and the first author, none of whom were involved in providing participants’ therapy.

##### The Primary Care Therapy Process Rating Scale (PCTPRS, Godfrey et al. 2007)

The PCTPRS was devised to examine adherence to CBT and counselling in an NHS setting. The PCTPRS comprises three sub-scales; one assessing the extent to which the therapist uses CBT techniques, one assessing the use of counselling and one to provide a measure of therapeutic alliance. We used the first two of these subscales but instead used the TPOCS-A (this was the preferred measure as, unlike PCTPRS, it has been developed specifically for children with anxiety) to measure therapeutic alliance. One session from all CBT and counselling participants was viewed and rated on the CBT subscale and on the counselling subscale. Items on the CBT subscale reference techniques uniquely characteristic of CBT, such as assigning homework and recognising cognitive errors, items on the counselling scale describe actions such as reflective listening and providing supportive statements. Although the interventions have some common elements, if therapists are adhering to the interventions, then CBT sessions should score highly on the CBT scale and low on the counselling scale, and conversely, counselling sessions should score low on the CBT scale and high on the counselling scale. Godfrey et al. (2007) report good inter-rater reliability and internal consistency for the scales of PCTPRS. For our study, Cohen’s Kappa (weighted) was 0.77 for the CBT scale of the PCTPRS and 0.46 for the counselling scale, regarded as ‘substantial’ and ‘moderate’ respectively (Landis and Koch 1977).

##### Therapy Process Observational Coding System—Alliance Scale (TPOCS-A; McLeod and Weisz 2005)

A measure of therapeutic alliance is considered important to include to assess young people’s therapy for a number of reasons: Firstly, being dependent on parents, they may not be attending entirely through their own volition and parent and child may not always agree on treatment goals (McLeod and Weisz 2005). Secondly, making meaningful connections with others is one of the obvious difficulties that a client group with ASD faces. Therefore, establishing an alliance with both parent and child presents a distinct challenge to therapists. TPOCS-A has been developed for children and adolescents receiving treatment for anxiety and has been reported to demonstrate good interrater reliability, internal consistency, and convergent validity (Fjermestad et al. 2012; McLeod and Weisz 2005). Inter-rater reliability between this study’s three raters using Cohen’s Kappa (weighted) was 0.75, deemed ‘substantial’ (Landis and Koch 1977).

### Interventions

MASSI (White et al. 2010) includes CBT for anxiety reduction and supplementary strategies targeting social skill deficits, themselves commonly a source of anxiety for adolescents. The non-directive supportive counselling offered in the study aimed to build rapport and encourage the expression of feelings using reflective listening, supportive statements, clarification and appropriate empathy. The counselling intervention dealt with anxiety as and when the client raised it, in a supportive manner, but with no focus on physical symptoms or cognitions. If, for example, anxiety was raised in terms of relationships, then the relationship would be explored, but not symptoms. All participants were offered 12 individual sessions (plus one booster session if needed) and five group sessions for both the CBT and the counselling arms.

### Procedure

Individuals meeting initial screening criteria i.e., clinician diagnosis of ASD and anxiety disorder, were invited to participate by their treating clinician. Following parental and child consent, ADOS, ADI-R and ADIS were completed to confirm diagnoses and baseline measures were then administered. Participants were offered as many sessions as they wished to complete all questionnaires. Generally completion took around two to three separate sessions face-to-face with a blinded assessor in an interview room within one of the participating clinics. Participants were asked to nominate a teacher or teaching assistant at their school, who they felt knew them well. Nominated teachers were approached by the research team and invited to participate. Interviews with teachers took place face-to-face at schools, also with a blinded assessor. Initial baseline data was collected not >4 weeks before treatment commencement. After baseline data collection, participants were allocated into one of the treatment arms. Post-test measures were collected within 4 weeks of treatment completion and follow-up assessments within 12 weeks.

### Ethical Considerations

**Fig. 1:**
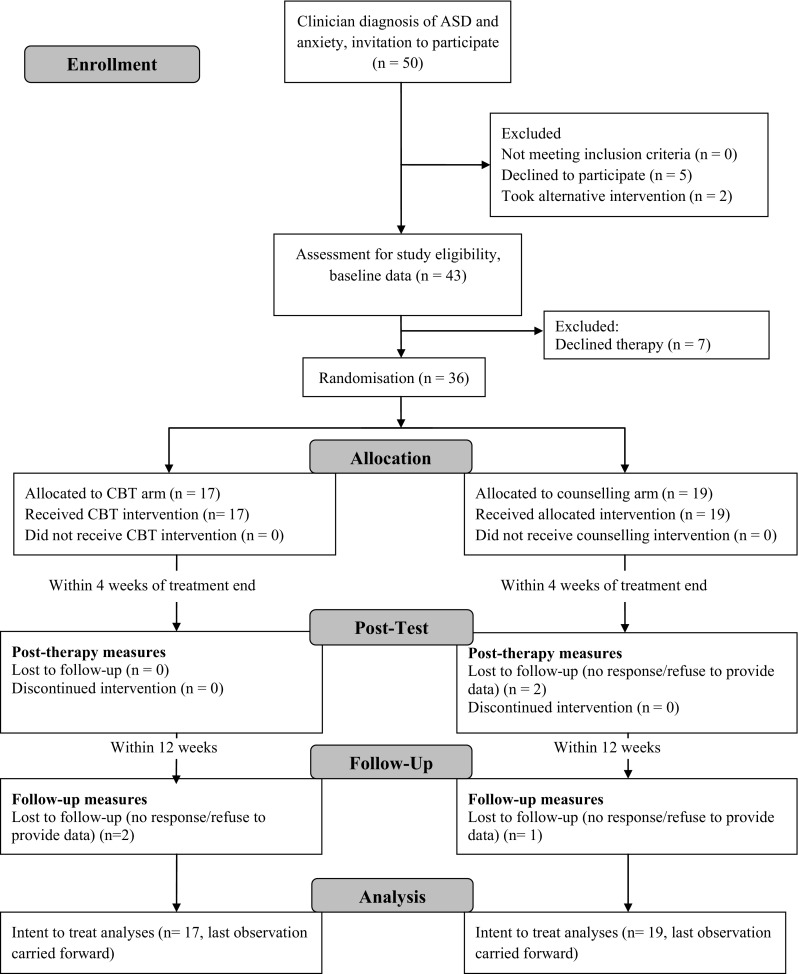
CONSORT 2010 flow diagram for study trial

A favourable opinion was received from the National Research Ethics Committee (East of England REC 11/EE/0285). All young people participating and their parents and teachers provided written consent after receiving full details (see Fig. 1). Participants were free to withdraw at any time without giving explanation. Participants did not pay for therapy nor did they receive any payment for participation.

### Analysis Plan

The primary aim of this study was to compare the effectiveness of CBT against counselling for the treatment of anxiety and accordingly, ADIS and CASI-anx, measures of anxiety, were designated primary outcome measures. A secondary aim was to compare outcomes for social skills between these two interventions as the CBT package included a supplementary social skills component; thus, SRS was included as a secondary measure.

Analyses were conducted on an intention-to-treat (ITT) basis; Fig. 1 illustrates participant characteristics of samples. Attrition was dealt with using a ‘last observation carried forward (LOCF)’ procedure. That is, baseline data was carried forward for the participants who dropped out and substituted for outcome data to allow the participants’ data to remain in the analyses. LOCF procedure is generally applied to ITT analyses, to limit potential bias in the form of analysis of treatment completers only.

Clinician severity ratings (CSRs) for ADIS range from 0 to 8, and a continuous score was generated for each participant for separation anxiety, social anxiety, specific phobia and generalised anxiety. Analysis of covariance (ANCOVA) was used to test differences in these CRS scores between the CBT and counselling interventions at immediate post-test and 12 week follow-up, with pre-intervention CSRs as the covariate. CASI-anx and parents’ and teachers’ SRS ratings were similarly analysed with pre-intervention ratings as the covariate. A dichotomous score was also generated for each participant indicating whether or not they met the diagnosis for separation anxiety, social anxiety, specific phobia or generalised anxiety (i.e., CSR threshold score 4 or more) and Fisher’s exact test was used to test for between-group differences. For the PCTPRS, TPOCS-A and attendance of session data, distributions were non-normal and non-parametric statistics (Mann–Whitney) were used for between-participants comparisons. Effect sizes (*r*) were calculated for all analyses, for non-parametric statistics *r* was calculated as proposed by Rosenthal and Rubin (2003). Consideration of effect sizes follows Cohen (1988) such that 0.1 is regarded as ‘small’, 0.3 is ‘medium’ and 0.5 or over is ‘large’.

## Results

### Participation and Attrition

Fifty patients were invited to participate. Of these, five declined as they felt unable to commit to the number of sessions. A further two expressed initial interest but subsequently preferred to take up interventions offered via schools instead. Forty-three patients were then assessed with ADI-R, ADIS and ADOS; after this point, a further seven declined to continue. Reasons for withdrawal at this point were: one felt she had progressed substantially since assessment and felt the intervention would no longer be of benefit, one experienced a bereavement so the timing of the intervention was not right, the parents of two young people felt that they could not attend parent sessions with work commitments and in three cases parents were keen to participate but the young person was unwilling. This therefore left 36 participants to be randomised; 17 were allocated to the CBT arm and 19 to the counselling arm (see Fig. 1).

For teacher-completed measures, response rates were poorer than for the adolescents and their parents. Although at baseline we were able to obtain completed questionnaires for 100% of nominated teachers for young people in full-time education (seven of our sample had left full-time education at 16), by 12-week follow-up attrition was more than 50% despite several reminders.

### Attendance of Intervention Sessions

For CBT, the mean number of individual sessions attended was 9.06 (*SD* 2.51) and for counselling 11.71 (*SD* 1.06), this was a significant difference (Mann–Whitney *U* = 32.5, *p* = .02). There was, however, no significant difference in number of participants attending at least three of the five group sessions; 11 of 17 CBT participants (61%), 9 of 19 counselling participants (47%, Chi square = 0.95, *p* = .32).

### Pre-treatment Comparability of Participants

Analyses were conducted to establish comparability of participants in the two arms at outset, no significant differences were found (see Table 1). Whilst high numbers of young people met the criteria for social and generalised anxiety and specific phobias, few reached the clinical threshold for separation anxiety. Low numbers of OCD, panic disorder and PTSD were in accordance with entry criteria for the study.

**Table 1:** Baseline pre-treatment comparability of participants across demographic, diagnostic, and intervention usage variables

	CBT (*N* = 17)	Counselling (*N* = 19)	*p* Value^f^
Child gender (male)	10 (59%)	12 (63%)	.53
Child ethnicity			
White	16 (94%)	18 (95%)	.74
Mixed ethnicity	1 (6%)	1 (5%)	n/a^e^
ADIS^a^ (meeting diagnosis)			
Separation anxiety	4 (22%)	5 (26%)	.73
Social anxiety	15 (83%)	16 (84%)	.55
Specific phobia	11 (61%)	17 (89%)	.25
Generalised anxiety disorder	15 (83%)	17 (89%)	.54
OCD	3 (16%)	1 (0.5%)	.58
PD/agor	0	0	0
PTSD	0	0	0
Medication usage			
All	3 (18%)	5 (26%)	.90
SSRI	1 (0.5%)	3 (15%)	.58
Tricyclic anti-depressant	0	1 (0.5%)	n/a^e^
Melatonin	1 (0.5%)	1 (0.5%)	n/a^e^
Methylphenidate	1 (0.5%)	0	n/a^e^

	*M (SD)*	*M (SD)*	*p* Value^f^
Child age (years)	14.94 (1.63)	15.56 (1.91)	.15
Conners^b^ (parent)	273.37 (50.36)	257.00 (45.74)	.32
Conners^b^ (teacher)	220.60 (43.38)	222.23 (40.80)	.91
CASI-anx^c^ (parent)	31.75 (12.05)	27.42 (10.66)	.26
SRS^d^ (parent)	167.06 (24.50)	159.68 (12.71)	.26
SRS^d^ (teacher)	146.86 (29.73)	128.52 (22.78)	.07

*OCD* Obsessive–compulsive disorder, *PD/agor* panic disorder with or without agoraphobia, *PTSD* post-traumatic stress disorder

^a^Baseline anxiety disorder: based on ADIS C/P Clinician Severity Rating of four or higher

^b^Conner’s Parent Scales and Conner’s Teacher Rating Scales, revised, short version, 1997

^c^Child and Adolescent Symptom Inventory-4 ASD Anxiety

^d^Social Responsiveness Scale, parent and teacher versions

^e^Not applicable, frequencies too low for meaningful statistics

^f^ *p* Value based on independent *t* test for continuous variables and Fisher’s exact test for categorical variables

### Treatment Fidelity

#### Primary Care Therapy Process Rating Scale (PCTPRS, Godfrey et al. 2007)

On the CBT subscale of the PCTPRS the mean score for CBT sessions was 25.88 (*SD* 11.98) and for counselling sessions was 9.62 (*SD* 8.50), a difference which was highly significant (Mann–Whitney *U* = 25.5, *p* < .0001).

On the counselling subscale of the PCTPRS the mean score for CBT sessions was 15.76 (*SD* 6.31) and counselling sessions 20.81 (*SD* 5.14), again a highly significant difference (Mann–Whitney *U* = 63.5, *p* < .001). The results suggest therefore, that the therapists delivering CBT and counselling sessions were faithful to the interventions.

#### Therapy Process Observational Coding System—Alliance Scale (TPOCS-A; McLeod and Weisz 2005)

Mean scores for the five therapists ranged from 29.95 (*SD* 5.42) to 39.00 (*SD* 1.41), with differences between the therapists being non-significant (Kruskal–Wallis, *H*(4) = 7.25, *p* = .07). Scores for individual questionnaire items for this study are given in Brown et al. (2015). It is noteworthy that, despite client group differences (McLeod and Weisz 2005 used typically-developing children aged 8–14, with anxiety), the scores obtained are largely similar, suggesting that the clinicians participating in our study were as successful in establishing positive relationships with clients with ASD as were clinicians in the original validation study for TOPCS-A. Across all therapists, the mean TPOCS-A score for the coded CBT sessions was 31.17 (*SD* 7.12) and for the counselling sessions was 31.81 (*SD* 6.56). This difference was non-significant (Mann–Whitney *U* = 120.0, *p* = .57).

### Outcome Measures

#### Clinician Severity Ratings (CSRs) and CASI-anx (Parent Rating)

CSR ratings on ADIS for the four different anxiety types (separation, social, generalised and specific phobias) are presented in Table 2. Differences by treatment (CBT versus counselling) for immediate post-intervention: CSRs were non-significant except for separation: *F*(1, 35) = 7.77, *p* = .01. Other results were social anxiety: *F*(1, 35) = 1.05, *p* = .31; specific phobia *F*(1, 35) = 0.43, *p* = .51; and generalised anxiety: *F*(1, 35) = 0.68, *p* = .41. Effect sizes were small bar separation which had a medium effect size. For 12 week follow-up all CSRs were non-significant: separation anxiety *F*(1, 35) = 2.40, *p* = .13; social anxiety: *F*(1, 35) = 1.69, *p* = .20; specific phobia: *F*(1, 35) = 1.57, *p* = .21; and generalised anxiety *F*(1, 35) = 0.64, *p* = .42. All effect sizes at 12 weeks were small. For CASI-anx (Table 2) results were also non-significant at immediate post-test *F*(1,35) = 1.88, *p* = .18, and at 12 week follow-up *F*(1, 35) = 0.95, *p* = .33, effect sizes again were small.

**Table 2:** Clinician severity ratings (CSRs) and CASI-anx (parent rating) for CBT versus counselling intervention (effect sizes >.30 italicised)

ADIS C/P^a^ subscale CSR	Baseline^b^		Post-test^b^		Follow-up^b^		Pre- to post-test^c^	Pre-test to follow-up^c^
	*M (SD)*		*M (SD)*		*M (SD)*		*r*	*r*
	CBT	Coun-selling	CBT	Coun-selling	CBT	Coun-selling		
Separation	1.52 (2.85)	1.47 (2.56)	2.11 (2.78)	0.47 (1.26)	2.35 (3.16)	1.15 (2.43)	.*43*	.13
Social	5.29 (2.14)	4.63 (2.16)	4.00 (2.78)	4.59 (1.46)	4.82 (2.69)	3.36 (2.89)	.17	.22
Specific phobia	3.29 (2.66)	4.57 (1.83)	3.17 (2.67)	3.00 (2.58)	3.94 (2.79)	3.52 (2.95)	.11	.20
Generalised anxiety	5.47 (2.18)	4.94 (1.87)	3.35 (2.71)	3.94 (2.57)	4.47 (2.91)	3.42 (2.98)	.13	.13
CASI-anx	31.75 (12.05)	27.42 (10.66)	27.93 (14.72)	22.26 (10.48)	27.28 (14.93)	20.13 (12.52)	.25	.18

*r*—effect size for CBT versus counselling contrast

^a^Anxiety Disorder Interview Schedule, child/parent combined score

^b^Intention to treat analyses, includes last observation carried forward

^c^ANCOVA with pre-intervention CSRs as covariate

#### Numbers of Participants Meeting Diagnoses

Diagnostic criteria measures (i.e., dichotomous measure, either above or below the threshold of CSR score 4) for ADIS showed no significant differences between CBT and counselling at either post-test or 12-week follow-up, with the exception of separation anxiety at post-test only (Table 3). This finding should be interpreted with caution; at outset, only nine participants of the entire sample met diagnostic criteria for this form of anxiety.

**Table 3:** Numbers of participants meeting diagnoses at baseline, post-test and 12-week follow-up

	Baseline		Post-test		*p* Value	Follow-up		*p* Value
	CBT	Coun-selling	CBT	Coun-selling		CBT	Coun-selling	
Meeting ADIS diagnosis	Yes^a^	Yes^a^	Yes/no^b^	Yes/no^b^		Yes/no^b^	Yes/no^b^	
Separation	4	5	4/0	1/4	.*04*	4/0	2/3	.11
Social	15	16	11/4	15/1	.14	12/3	9/7	.15
Specific phobia	11	17	8/3	9/8	.17	9/2	11/6	.29
Generalised anxiety	15	17	9/6	13/4	.26	12/3	10/7	.18

*p* Value, Fisher’s exact test, CBT versus counselling <.05 italicised

^a^Number of participants meeting diagnoses for at baseline

^b^Number of participants meeting diagnosis for ADIS/no longer meeting diagnosis

SRS ratings are presented in Table 4. In the analyses both parent- and teacher-ratings for Social Communication and Social Motivation subscales differences by treatment (CBT versus counselling) for immediate post-test were non-significant; parent, social communication *F*(1, 33) = 2.04, *p* = .16; parent, social motivation *F*(1, 33) = 3.49, *p* = .07; teacher, social communication *F*(1, 32) = 0.01, *p* = .93, teacher, social motivation *F*(1, 32) = 0.20, *p* = .66. At 12-week follow-up, the picture was similar, with no significant differences and very small effect sizes. Parent, social communication *F*(1, 26) = 1.97, *p* = .17; parent, social motivation *F*(1, 26) = 0.75, *p* = .39; teacher, social communication *F*(1, 11) = 0.65, *p* = .43; teacher, social motivation *F*(1, 11) = 0.12, *p* = .73. Caution should be exercised for the teacher follow-up result as loss at 12 weeks was over 50%.

**Table 4:** SRS parent and teacher ratings for social communication and social motivation subscales for CBT versus counselling intervention (effect sizes >.30 italicised)

SRS^a^	Pre-test^b^		Post-test^b^		Follow-up^b^		Pre- to post-test^c^	Pre-test to follow-up^c^
	*M (SD)*		*M (SD)*		*M (SD)*		*r*	*r*
	CBT	Coun-selling	CBT	Coun-selling	CBT	Coun-selling		
Parent rating Social com^d^	110.50 (15.30)	107.52 (8.68)	99.92 (14.87)	103.85 (10.12)	97.78 (15.52)	103.12 (11.16)	.27	.26
Parent rating Social mot^e^	110.06 (8.88)	102.31 (9.83)	101.35 (9.67)	106.92 (6.79)	103.85 (10.85)	98.50 (11.39)	.*35*	.16
Teacher rating Social com^d^	78.93 (10.83)	75.76 (8.81)	80.10 (7.10)	78.55 (11.87)	76.10 (5.96)	70.33 (5.85)	.02	.23
Teacher rating Social mot^e^	78.93 (10.78)	72.82 (7.41)	78.40 (6.99)	72.00 (9.06)	78.75 (9.89)	68.66 (6.31)	.10	.10

*r*—effect size for CBT versus counselling contrast

^a^Social Responsiveness Scale

^b^Intention to treat analyses, includes last observation carried forward

^c^ANCOVA with pre-intervention SRS values as covariate

^d^SRS social communication subscale

^e^Social motivation subscale

## Discussion

In summary, no significant differences were found between the MASSI CBT intervention and the counselling intervention on any of the measures taken with the sole exception of separation anxiety (ADIS) at immediate post-test. This separation anxiety result does however need to be treated with extreme caution, as only 9 out of 36 (25%) of the sample met clinical criteria for this form of anxiety. Measures taken included parent- and child- report for ADIS, regarded as the ‘gold standard’ anxiety measure (Ung et al. 2014) and CASI-anx, devised specifically to measure anxiety in children with ASD (Sukhodolsky et al. 2008) as well as both parent- and teacher-reports of social disabilities (SRS, Constantino and Gruber 2005). These results are consistent with previous findings for anxiety treatment for adults (Cape et al. 2010) and similarly, James et al. (2015) found no differences in their review between CBT treatment and active controls for children. It must be acknowledged however, that few studies have undertaken these comparisons and indeed, we responded to calls from a number of authors for comparisons of CBT comparisons with alternative treatments (e.g., James et al. 2015; Storch et al. 2013; Sukhodolsky et al. 2013).

An important point to stress is that whilst there were no significant differences between CBT and counselling in this study, this does *not* mean that these interventions did not *individually* produce improvements in the participants (see Tables 2, 3, 4, also both therapies have separately previously shown effectiveness for anxiety in trials against wait-lists, James et al. 2015). The results show that neither treatment was indicated as superior to the other by our measures of anxiety and social skills.

An interesting difference between the two interventions concerns attendance for individual sessions, with attendance at counselling sessions being significantly higher. Unfortunately, previous trials in this area have rarely reported attendance, making comparison of this aspect of our study with others difficult. Although there has been little investigation of the optimal number of sessions or hours for anxiety treatments, Reynolds et al.’s (2012) meta-analysis of psychotherapy interventions for anxiety in children did compare effect sizes for interventions offering different numbers of treatment hours. They reported that moderate to large treatment effects were associated with nine or more hours of treatment. Mean attendances for counselling and CBT were 11.71 and 9.06 respectively (out of 12) in our study, suggesting that both treatments did reach optimal amounts. However, counselling may have been a more appealing treatment and as a result the significantly higher dosage received in this treatment arm may obscure differences between counselling and CBT. Nevertheless, it has to be remembered that whilst CBT has been shown to be superior to passive treatments (such as wait-list) in large numbers of studies, it has *not*, as discussed in our introduction, shown superiority against active treatments (James et al. 2015). Study results provide no indication as to why counselling may have been a more attractive treatment, but as scores for therapeutic alliance for the two therapies were very similar, it is unlikely that this is where the difference lies, furthermore, satisfaction rates have been high for both counselling and CBT in previous research (Bower et al. 2011). This was one of the first studies to include a measure of therapeutic alliance with children with ASD. It is noteworthy that despite the purported challenges in establishing positive relationships with children with ASD, scores obtained by our clinicians were similar to those in McLeod and Weisz’s (2005) study, thus emphasising the importance of making therapies available to this client group.

Some consideration of our participants is warranted, as we had a somewhat higher than usual proportion of girls in our sample. Probable reasons for this relate to the mean age of our sample, at 15.25 years, this was older than all other studies conducted thus far with children with ASD (see Ung et al. 2015). Girls are typically diagnosed with ASD later than boys and furthermore, anxiety is more common in girls with ASD than in boys with ASD who instead tend to exhibit more externalising problems (Loomes et al. 2017). There is also evidence of a diagnostic bias (Loomes et al. 2017), we were fortunate to work with an experienced clinical team with awareness of ASD symptoms in girls who thus may have been less subject to this diagnostic bias.

### Strengths and Limitations

A limitation of our study is its small sample size and it should therefore be considered a pilot. Sample sizes for previously published trials for the treatment of anxiety by CBT for children with ASD range from 12 to 71 (Ung et al. 2015) so, as yet, no large scale definitive RCTs have been completed. It is possible that larger sample sizes would detect subtler differences and smaller effect size differences between CBT and other interventions. However, it should be remembered that our study addressed a number of issues that limited previous studies: First, outcome measures were taken from a variety of reporters; we obtained both parent- and child-report for ADIS and social responsiveness (SRS) was observed by both parents and teachers separately. Teachers are often more accurate judges of children’s social difficulties, particularly with peers, by comparison to parents who may be anxious and over-estimate their children’s difficulties (Hallett et al. 2013). Second, previous studies (e.g., Storch et al. 2013; Wood et al. 2015) have followed up only active (i.e., CBT only) participants beyond immediate post-test, whereas we followed both trial arms up to 12 weeks. Third, stringent fidelity measures were included to ensure adherence to both treatments in addition to a measure of therapeutic alliance to detect possible therapist variations between the interventions, very few intervention studies with ASD clients have included these checks. Whilst the conclusions advanced here are tentative, these strengths add some support.

## Conclusion

Although this study is the first to compare CBT against a counselling intervention for the treatment of anxiety specifically in children with ASD, its findings are consistent with previous findings for anxiety treatment for children in general; James et al. (2015) found no significant differences between CBT interventions and non-CBT *active control* treatments (although significant differences have consistently been found between CBT and *passive* control groups). As regards children with ASD, in the only trial to date to compare CBT to an alternative active intervention, Sung et al. (2011), found no significant differences. For clients who do not wish to receive CBT, or have not found it to be effective for their needs, further research may usefully investigate counselling as an alternative therapy.
